# Efficacy of Remineralizing Agents for Prevention of Microhardness Reduction and Change in Mineral Content of Enamel in Anterior Primary Teeth after Exposure to Iron Drop 

**DOI:** 10.30476/dentjods.2024.100763.2247

**Published:** 2025-06-01

**Authors:** Aneseh Sadat Tabatabaei Rad, Sara Tavassoli-Hojjati, Reyhane Sadat Hoda, Saba Aghaei

**Affiliations:** 1 General Dentist, School of Dentistry, Islamic Azad University of Tehran, Tehran, Iran.; 2 Dept. of Pediatric, Faculty of Dentistry, Tehran Medical Sciences, Islamic Azad University, Tehran, Iran.

**Keywords:** Dental Enamel, Hardness, Iron Compounds, Scanning Electron Microscopy, Tooth Remineralization

## Abstract

**Background:**

Some alterations in the enamel structure of primary teeth have been reported following iron drop consumption. The efficacy of different remineralizing agents for this problem is still challenging.

**Purpose:**

This study aimed to assess the efficacy of remineralizing agents for prevention of microhardness reduction and change in primary enamel mineral content after exposure to iron drop.

**Materials and Method:**

In this experimental study, 36 sound primary anterior teeth were assigned to four groups of control, casein phosphopeptide amorphous calcium phosphate (CPP-ACP), fluoride varnish (FV), and CPP-ACP+FV (MI varnish). The baseline microhardness of the teeth was first measured by Vickers hardness tester using 50 g load for 10 seconds. Next, the microhardness was measured after remineralizing agents were applied on the specimens. After the application of iron drop for 5 minutes, and pH cycling for 10 days, the final microhardness of specimens was registered. Specimens were also subjected to energy dispersive X-ray spectroscopy (EDS) in the control group, after remineralization and after the application of iron drop. Data were analyzed by one-way ANOVA, Tukey’s test, and Tamhane’s test.

**Results:**

The final microhardness was significantly higher in all remineralized groups compared to the control group (*p* Value= 0.003). The final microhardness was significantly higher in MI varnish compared to FV (*p*= 0.027), CPP-ACP (*p*= 0.03), and control (*p*< 0.0001) groups. According to EDS, calcium (Ca) and ferric (Fe) content were significantly different between the remineralizing groups and control groups in the final step (*p*< 0.00001).

**Conclusion:**

Application of CPP-ACP, FV, and MI varnish prior to iron drop exposure can improve microhardness. MI varnish had significantly higher efficacy for this purpose than the other two remineralizing agents.

## Introduction

The prevalence of iron deficiency was 18% to 38% in Iranian children under 5 years of age in 2004 [ 1 - 5 ]. In addition, the adverse effects of iron deficiency anemia on young children often continue over years, and decrease their cognitive function, and physical and behavioral performance and adverse effect on different part of body such as central nervous system, gastrointestinal, and immunity [ 6 ]. Iron drops are prescribed after 6th month to 2 years to prevent iron deficiency [ 1 , 6 ]. The majority of iron supplements contain citric acid to improve their flavor and taste; however, citric acid decreases the pH and increases the solubility of tooth structure [ 5 ], resulting in dental erosion [ 7 ] and microhardness reduction [ 1 , 3 ]. In addition, increased enamel surface roughness results in dark discoloration and development of dental caries [ 1 - 4 , 8 ]. 

It has been demonstrated that casein phosphopeptide amorphous calcium phosphate (CPP-ACP) can enhance remineralization and could prevent dental caries [ 9 ]. CPP-ACP is a bioactive agent derived from milk protein. Casein phosphopeptide (CPP) binds to dental plaque, soft tissue, and tooth and provides a super-saturated state of minerals in the enamel by stabilization and localization of amorphous calcium phosphate (ACP) [ 10 ]. As a result, mineral loss due to acid attacks would be reduced [ 11 ]. The cariostatic and remineralizing properties of fluoride have been well documented [ 11 ]. The synergistic effects of CPP-ACP and fluoride for prevention of demineralization have been previously reported as well [ 12 - 14 ]. A previous study showed that simultaneous application of CPP-ACP and fluoride varnish (FV) for the prevention of enamel demineralization was more effective than monotherapy with each of them alone [ 13 ]. Another study revealed that MI varnish containing CPP-ACP and fluoride significantly decreased demineralization compared with each of them alone [ 15 ]. Nonetheless, the efficacy of these bioactive remineralizing agents in the prevention of primary enamel microhardness reduction due to exposure to iron drop has not been previously investigated. Thus, this study aimed to assess the efficacy of remineralizing agents for prevention of microhardness reduction and change in mineral content of enamel in anterior primary teeth following exposure to iron drop. 

## Materials and Method

In this experimental study (ethical approval code: IR.IAU.DENTAL.REC.1400.043), the minimum sample size was calculated to be 9 in each of the four groups according to a study by Mohammed *et al*. [ 16 ] assuming α=0.05, β=0.2, mean standard deviation of 1.5, and effect size of 0.62 using one-way ANOVA power analysis feature of SPSS 26. 

A total of 36 sound primary maxillary anterior teeth with no caries, cracks, fracture, restoration, or hypoplasia in their crowns were included in this study (with satisfaction of parents) [ 1 - 3 , 17 - 19 ]. The teeth were inspected under a stereomicroscope (LV-TV, Nikon, Japan) at 40× magnification to ensure absence of cracks. The maximum time interval between tooth extraction and the experiment was 3 months [ 1 ]. All teeth were immersed in 0.5% chloramine T solution for one week and stored in distilled water at 4°C until the experiment [ 20 ]. The enamel microhardness of primary teeth was measured before and after exposure to iron drop and also before the application of remineralizing agents by a Vickers hardness tester (Wilson Hardness Tukon 1202, Buehler, USA) [ 19 , 21 ]. The specimens underwent the following eight steps as follows.

### Specimen preparation

The entire crown surfaces of all 27 primary maxillary anterior teeth were coated with nail varnish (Natural Purl, Iran) except for a 4×4mm window in the labial surface [ 21 ]. 

### Baseline measurement of microhardness

The teeth were mounted in light-cure acrylic resin (Megadent, Megatray, Germany) such that their lingual surface was mounted in acrylic resin while their labial surface was exposed (facing out) [ 18 ]. To obtain a smooth enamel surface for indentation, the labial surface was polished with 800, 1000, and 2000-grit silicon carbide abrasive papers (Smirdenx, Greece) under running water [ 3 , 5 , 21 - 22 ]. Each abrasive paper was used with 90-degree rotation compared with the previous abrasive paper for each surface to obtain a smooth surface for indentation and measurement of microhardness. The microhardness of specimens was measured by a Vickers hardness tester [ 1 , 18 , 22 ]. The labial surface of the teeth was assessed, and the indenter applied 50 g load for 10 seconds to create a square-shaped indentation [ 18 ]. The Vickers hardness number (VHN) of each specimen was measured at three points as such, and the mean of the three values was recorded as the VHN of the respective specimen [ 1 , 18 ]. Teeth with a VHN value of 239 to 478 were selected for this study [ 18 ]. The teeth were then immersed in distilled water at room temperature [ 3 , 20 , 22 ]. 

### Remineralization 

The teeth were randomly assigned to three groups by simple randomization using a computer-generated table of random numbers, such that 9 teeth were assigned to each group of CPP-ACP, FV, and MI varnish (a combination of CPP-ACP and FV).

In each group, CPP-ACP (Tooth Mousse/GC, USA), FV (Flouro Dose/centric, USA), and MI varnish (GC, USA) were applied on the window created on the buccal surface for 4 hours. Next, they were removed by a cotton swab dipped in acetone, and the tooth surface was rinsed with distilled water [ 23 ]. 

**Figure 1:**
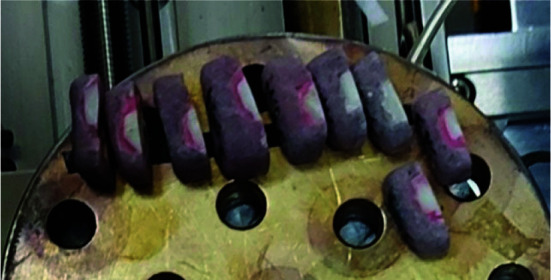
Samples halves

**Figure 2:**
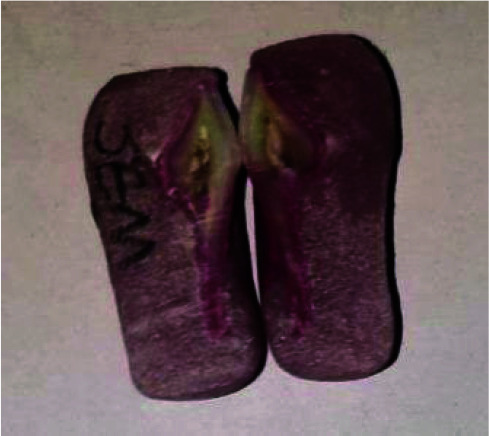
Another aspect of sample halves

After the application of remineralizing agents, the teeth were immersed in artificial saliva with the composition of 1.5 mM CaCl_2_, 0.9 mM NaH_2_PO_4_, and 0.15 M KCl with a pH of 7 for 24 hours [ 21 ]. Next, each tooth was split in half labiolingually along the longitudinal axis and at the center of the created window using a diamond disc (Prodont Holliger, France) (Figures 1-2). One-half of each tooth underwent the next steps and the other half was used for assessment of mineral content by energy dispersive X-ray spectroscopy (EDS).

### Measurement of secondary microhardness

Tooth halves subjected to remineralizing agents were removed from the artificial saliva and rinsed with distilled water [ 3 , 16 , 22 ]. Their microhardness was then measured again blindly by a Vickers hardness tester. 

### Exposure to iron drop

Acetone was used to remove nail varnish from the tooth halves. They were then exposed to iron drop (Irofant ferrous sulfate, Kharazmi, Iran) for 5 minutes at 37°C in a shaker incubator (Shin Saeng, Korea) [ 1 , 3 , 18 , 22 , 24 ]. This iron drop contains 125.1 mg ferrous sulfate heptahydrate per each 1 mL (25 drops) and 25 mg sodium saccharine (as sweetener) [ 3 , 22 ]. 

### The pH cycling

Tooth halves underwent remineralization-demineralization cycles. The composition of demineralizing solution included 2.2 mM CaCl_2_, 2.2 mM NaH_2_PO_4_, 0.05 M acetic acid, and 1 M KOH at a pH of 4.4, while the formulation of remineralizing solution included 1.5 Mm CaCl_2_, 0.9 Mm NaH_2_PO_4_, 0.15 M KCl, and 1 ppm NaF with a pH of 7 [ 23 ]. The teeth were immersed in demineralizing solution for 6 hours and in remineralizing solution for 18 hours at 37°C on a daily basis for 10 days [ 23 ]. The solutions were refreshed daily [ 4 ]. 

### Measurement of final microhardness 

The microhardness of the tooth halves was measured again blindly by a Vickers hardness tester. The change in microhardness of each tooth was calculated quantitatively by comparison with the baseline values. 

### Measurement of mineral content

**Figure 3:**
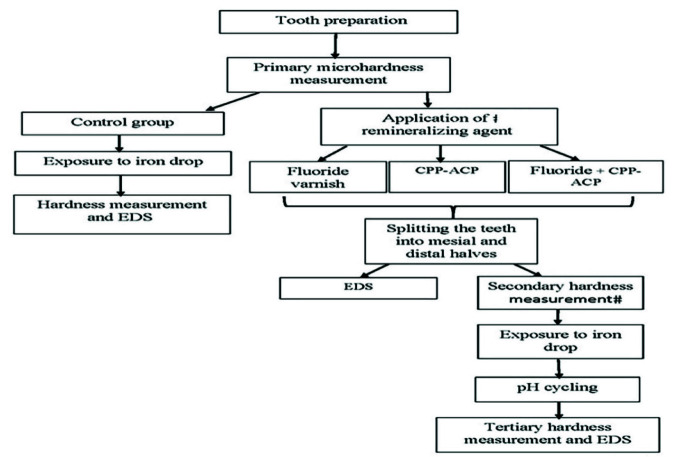
Study design flow diagram

All tooth halves were rinsed with deionized water and prepared for the measurement of mineral content by energy dispersive X-ray spectroscopy (EDS) (EDS/mapping–BRUKER XFlash 6.10, Netherlands). Each tooth half was mounted on a piece of copper, and subjected to EDS. The content of calcium, phosphorous, fluoride, and iron based on their atomic number was determined according to the diagram peaks shown by the software (Figure 3) [ 21 ]. 

### Statistical analysis

Data were analyzed by the SPSS software version 26 (SPSS Inc., Chicago, IL, USA) using one-way ANOVA, Tukey’s test, and Tamhane’s test at *p*< 0.05 level of significance. 

## Results

**Figure 4:**
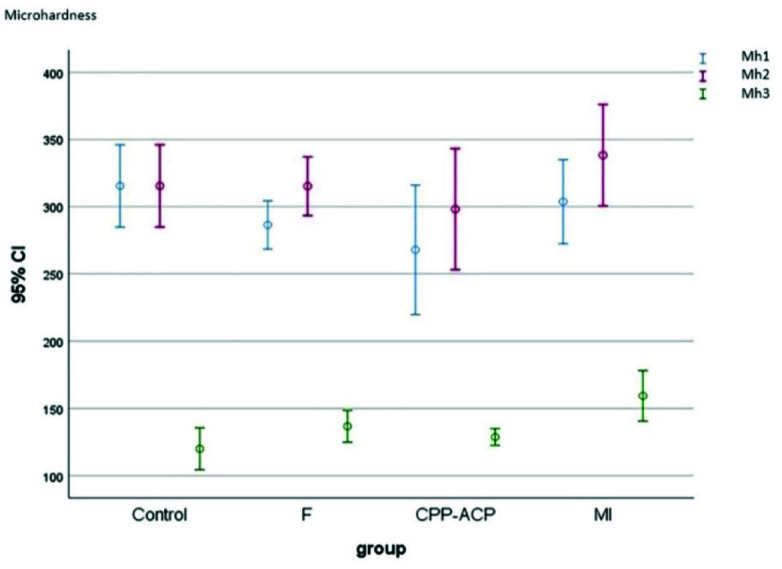
Microhardness of the groups at three steps

Table 1 and Figure 4 present the baseline, secondary, and final microhardness of the groups. Considering the normal distribution of data as confirmed by the Kolmogorov-Smirnov test (p> 0.05), one-way ANOVA was used for the comparisons, which showed a significant difference among the baseline, secondary, and final microhardness within each group (*p*< 0.0001), such that the microhardness at the second step (after the application of remineralizing agent) increased by 10% in FV, 11.2% in CPP-ACP, and 11.3% in MI varnish group, compared with the first step. Also, the microhardness at the third step (after exposure to iron drop) decreased by 52% in FV, 51% in CPP-ACP, and 48% in MI varnish group, compared with the second step. This reduction was 63% in the control group that was only exposed to iron drop and did not receive remineralizing agents. 

**Table 1:** Mean Microhardness (VHN) and mineral content of the groups

Tests	Mean± SD Microhardness (Kgf/mm²)			*p* Value	Tukey HSD Mean± SD Mineral content (wt%)								*p* Value
Groups	Mh1^a^	Mh2^b^	Mh3^c^	Multiple Comparisons	Ca	CaH	P	PH	F	FH	Fe	FeH	Paired Samples Test
Control (iron)	315.4000 ± 39.80710	_	119.8667 ± 20.27042		65.3433 ± 2.44856	_	28.9078 ± 3.19174	_	0.3144 ± 0.55935	_	5.1522 ± 1.95029		_
FV	286.4222 ± 23.41654	315.2333 ± 28.39820	136.5889 ± 15.34784	Mh1-Mh2: >0.0001^*^	67.3267 ± 1.95630	69.7011 ± 1.09235	30.3856 ± 1.97467	30.0356 ± 1.01214	0.2567 ± 0.37829	0.1511 ± 0.21292	2.0311 ± 0.49245	0.1122 ± 0.11454	Ca-CaH^#^: 0.007*
				Mh1- Mh3: >0.0001*									P-PH^#^: 0.627
				Mh2- Mh3: >0.0001*									F-FH^#^: 0.467
													Fe-FeH^#^: >0.0001*
MI varnish	303.6556 ± 40.63423	338.2444 ± 49.08457	159.2444 ± 24.44673	Mh1-Mh2: >0.0001*	67.9722 ± 1.68534	72.1456 ± 2.29956	29.7989 ± 1.60973	27.4911 ± 2.14565	0.3867 ± 0.38846	0.2000 ± 0.30871	1.7422 ± 0.34734	0.1633 ± 0.13398	Ca-CaH: 0.002*
				Mh1- Mh3: >0.0001^*^									P-PH: 0.032^*^
				Mh2- Mh3: >0.0001^*^									F-FH: 0.098
													Fe-FeH: >0.0001^*^
CPP-ACP	267.9111 ± 62.64398	298.1222 ± 58.60955	128.6222 ± 8.09966	Mh1-Mh2: >0.0001^*^	68.3944 ± 1.33720	70.2267 ± 0.86324	29.5100 ± 1.39556	29.5100 ± 0.99911	0.1144 ± 0.12885	0.1611 ± 0.32402	1.9811 ± 0.41664	0.1033 ± 0.12217	Ca-CaH: 0.013^*^
				Mh1- Mh3: >0.0001^*^									P-FH: 0.999
				Mh2- Mh3: >0.0001^*^									F-FH: 0.716
													Fe-FeH: >0.0001^*^
p value	0.133	0.331	<0.0001*		0.009^*^	-	0.541	-	0.520	-	>0.0001^*^	-	

- Mineral content was measured after the application of the iron drop in the control group and after the application of remineralizing agents+iron drop in the study groups

- As remineralizing agents were not applied in the control group, Mhs2 was not measured in this group.

*: significant

a: Mh1: first microhardness

b: Mh2: microhardness after consuming remineralizing agent

c: Mh3: microhardness after using iron drop

#: CaH/FeH/PH/FH: Ca, Fe, P, F content in dental halves that were only under the effect of remineralizing agent

**Figure 5:**
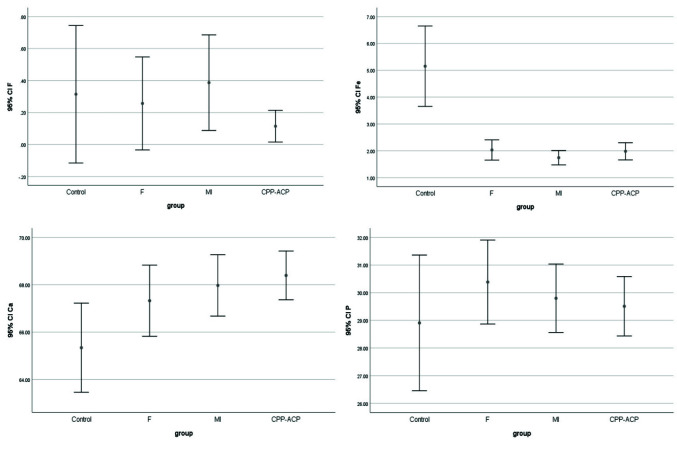
Ca, F, P and Fe contents of the study groups as shown by energy-dispersive X-ray spectroscopy (EDS)

One-way ANOVA showed a significant difference in the amount of Ca (*p*= 0.009) and Fe *p*< 0.0001) ions on the external tooth surface in different groups. The highest calcium content was noted in CPP-ACP group and the lowest in the control group. Also, the highest iron content was found in the control group and the lowest in MI varnish group. Considering the variance and data distribution in each group, pairwise comparisons of the groups were conducted by the Tukey’s test for the calcium content and by the Tamhane’s test for the iron content. The Tukey’s test showed that the difference in calcium content was significant between the control and MI varnish (*p*= 0.030), and control and CPP -ACP (*p*= 0.009) groups. The Tamhane’s test revealed significant differences between the control group with CPP-ACP (*p*= 0.007), FV (*p*= 0.007), and MI varnish (*p*= 0.004) groups regarding the iron content. The elemental content of the two halves of each tooth (the half subjected to remineralizing agents and iron, and the other half subjected to remineralizing agents) was compared within each group by paired sample t-test. In total, the iron content was higher and the calcium content was lower in tooth halves, subjected to iron drop and remineralizing agents, compared with other halves, subjected to remineralizing agents alone (Table 1 and Figure 5). 

## Discussion

As consuming iron drop supplements leads to demineralization and reduction of primary enamel microhardness among children [ 11 ], this study aimed to assess the efficacy of CPP-ACP, FV, and MI varnish for the prevention of microhardness reduction and mineral content change of enamel in anterior primary teeth following exposure to iron drop. 

The present study found no significant difference in enhancement of microhardness among the tested remineralizing agents (*p*> 0.05). MI varnish and CPP-ACP create a super-saturated state of calcium and phosphorus at the tooth surface, which enhances enamel remineralization and limits demineralization and mineral loss in the process of caries development [ 13 ]. In addition, FV results in formation of calcium fluoride (CaF_2_) deposits and increases enamel resistance, enhances remineralization, and subsequently improves the microhardness of the teeth [ 25 ]. 

Our results revealed that iron drop significantly decreased the surface microhardness in all study groups, which was in accordance with the studies of Tabari *et al*. [ 1 ], Mehran *et al*. [ 3 ], and Pasdar *et al*. [ 18 ]. Also, Abbasi *et al*. [ 26 ] evaluated the effect of iron drop and multivitamins on microhardness of primary teeth and found that Kharazmi iron drop caused 43.47% reduction in primary enamel microhardness. Iron drop decreases the microhardness by two mechanisms. It contains citric acid with a pH of 2.54 to 4.68 which is lower than 5.5, and increases the solubility and dissolution of hydroxyapatite, and development of dental erosion [ 26 ]. Acidic compounds are added to medications and supplements as a buffer to preserve chemical stability, control cohesion, and enhance physiological compatibility, solubility, and taste, and subsequently improve their acceptance by patients [ 27 - 28 ]. Moreover, when enamel is incubated with iron salts, ferric sulfate deposits will form on the enamel surface due to interaction of iron ions with insoluble phosphates. This composition has a weaker structure than calcium phosphate and decreases the microhardness of the teeth [ 29 - 31 ]. Since primary enamel is less mineralized than permanent enamel, it is more susceptible to dental erosion and discoloration following the consumption of compounds with a low pH [ 32 ]. 

The present study indicated that the application of remineralizing agents on primary teeth prevented the reduction in microhardness following exposure to iron drop to some extent. MI varnish was significantly more effective than CPP-ACP and FV for this purpose (*p*< 0.0001). Moreover, in some other studies comparing the efficacy of these products in prevention of erosion, the demineralization prevention potential of MI varnish was higher than that of FV and CPP-ACP [ 13 , 15 ]. Application of CPP-ACP along with fluoride has a synergistic effect on enamel remineralization and formation of stabilized amorphous calcium phosphate fluoride, which results in increased participation of fluoride ions and increased concentration of available calcium and phosphate ions [ 13 ]. To date, only one study has been conducted on prevention of adverse effects of iron drops. Tabari *et al*. [ 1 ] used nano-hydroxyapatite and chitosan remineralizing agents prior to exposure of teeth to iron drops. They reported the minimal efficacy of this remineralizing compound in prevention of microhardness reduction, and showed that the erosive potential of iron drop was much higher than the remineralizing effect of this compound. 

The second objective of the present study was to assess the mineral content of enamel of primary teeth after the application of remineralizing agents and exposure to iron drop. The results of EDS analysis showed an increase in calcium content of primary enamel in the final step in MI varnish, CPP-ACP, and FV groups. CPP-ACP and MI varnish remineralizing agents have CPP complexes that increase the mineral content of the teeth. CPP competes with calcium in binding to plaque calcium binding sites, limiting mineral loss in the process of caries development, and creating a super-saturated state of calcium and phosphorous close to the tooth surface, resulting in enhancement of remineralization [ 33 ]. 

Moreover, tooth halves subjected to iron drop and remineralizing agents had higher iron content and lower calcium content that the other halves subjected to remineralizing agents alone. Kharazmi iron with a pH of approximately 1.9 [ 24 ] can cause demineralization and enamel erosion. To overcome this acidic pH, the tooth structure releases calcium and phosphate, and becomes porous and Iron ions react with the phosphates [ 18 , 33 ]. In a study by Salman *et al*. [ 21 ] evaluating the effect of remineralizing agents on demineralized lesions used EDS analysis, MI varnish increased the mineral content significantly more than FV. 

In the present study, the fluoride element was also analyzed in EDS but no significant difference was noted in this respect among different groups. Similarly, in the study of Vicente *et al*. [ 35 ] on prevention of enamel demineralization by fluoride varnishes, no significant difference was found regarding the amount of fluoride ions. They stated that the storage environment affects the amount and speed of release of fluoride. Perhaps the reason for the lack of difference in enamel fluoride levels between the groups is due to the topical application of fluoride, which initially leads to the formation of calcium fluoride on the enamel surface, and later enters the enamel structure. Since EDS analysis detects fluoride within the enamel structure, no difference in the amount of enamel fluoride was observed [ 25 , 34 ]. 

Evaluation of the efficacy of commonly used remineralizing agents in pediatric dentistry for prevention of microhardness reduction by Kharazmi iron drop for the first time was the main strength of the present study. Moreover, in addition to measurement of microhardness, EDS analysis was performed, and pH cycling was conducted to simulate the oral environment. Besides, tooth halves were used to compare remineralization plus iron drop and remineralization alone in the same tooth to eliminate the confounding effect of structural differences of the teeth on the results. The *in vitro* design of this study may limit the generalization of results to the oral environment. 

## Conclusion

Within the limitations of this study, the results showed that application of FV, CPP-ACP, and MI varnish remineralizing agents increased the primary enamel microhardness. Moreover, applying remineralizing agents prior to iron drop exposure prevented microhardness reduction by approximately 10%. MI varnish was significantly more effective than CPP-ACP and FV for this purpose. Since iron drop exposure significantly decreased the microhardness of primary enamel, CPP-ACP, MI varnish, and fluoride varnish were effective in prevention of reduction in mineral content following exposure to iron drop. 
